# Ruptured mesenteric desmoid-type fibromatosis without emergency surgery: A rare case report

**DOI:** 10.1016/j.ijscr.2021.106208

**Published:** 2021-07-17

**Authors:** Masahiro Tawada, Yuki Misao, Takuya Sugimoto, Hidenori Tanaka

**Affiliations:** Department of Surgery, Asahi University Hospital, 3-23, Hashimoto-cho, Gifu, Gifu 500-8523, Japan

**Keywords:** Mesenteric desmoid-type fibromatosis, Rupture, Without emergency surgery, Case report

## Abstract

**Introduction:**

Desmoid-type fibromatosis (DF) is a rare tumor that develops in the limbs, abdominal wall, and abdominal cavity. It is accounting for less than 3% of soft-tissue sarcomas and less than 0.03% of all neoplasms.

**Presentation of case:**

A 57-year-old man was diagnosed as acute peritonitis due to intra-abdominal tumor rupture. Since his systematic symptoms were relatively stable, gastrointestinal perforation was ruled out, the differential diagnosis of the tumor itself was difficult, and it was unclear resectable by emergency surgery, we started conservative treatment. After examinations, ileocolectomy was performed. Histopathological examination revealed spindle cells with collagenous fiber hyperplasia and immunohistochemical staining for β-catenin was positive, so we made a diagnosis of mesenteric desmoid-type fibromatosis (MDF).

**Discussion:**

The mechanism of DF development is suggested to be associated with hereditary diseases, mechanical stimuli, and a history of exposure to radiation appear to be involved as pathogenetic factors in sporadic development. Surgical resection is the first-line treatment for MDF, but the postoperative high local recurrence rate is problematic. Drug therapy and radiation therapy are selected for cases in which radical resection is not possible or for recurrent cases. However, the number of examined cases is small and sufficient evidence has not been accumulated for most treatment strategies, it is expected that the optimal treatment at the time of recurrence will be further verified by the accumulation of MDF.

**Conclusion:**

There are few reports of peritonitis caused by MDF rupture; emergency surgery can be avoided.

## Highlights

• Mesenteric desmoid-type fibromatosis (MDF) is rare tumor, difficult to diagnose because of nonspecific signs and symptoms.
• There are few reports of peritonitis caused by MDF rupture; emergency surgery can be avoided.
• Surgical resection is the first-line treatment for MDF.
• Local recurrence may occur due to tumor rupture of the abdominal cavity, so careful follow-up is essential.

## 1 Introduction

DF is a rare tumor that develops in the limbs, abdominal wall, and abdominal cavity. It is accounting for less than 3% of soft-tissue sarcomas and less than 0.03% of all neoplasms. The DF frequency of occurrence is 2.4 to 4.3/million. DF is more common among women and is classified into three types: extra-abdominal wall type affecting the head, neck and limbs (43%), abdominal wall type (49%), and intraperitoneal type (8%); and the intraperitoneal type is the least [1]. Furthermore, the intraperitoneal type can be divided into the mesenteric type (81%) and pelvic type (19%) [2], [3].

Although pathologically benign, it is classified as intermediate/locally aggressive and non-metastasizing in the new 2013 World Health Organization tumor classification because of its characteristics of invasive growth, local recurrence, and no distant metastasis. We report a case of MDF ruptured to cause peritonitis, and resection of the tumor was possible after conservative treatment, without emergency surgery.

This case report has been reported in accordance with the SCARE Criteria [4].

## 2 Presentation of case

**Fig. 1:**
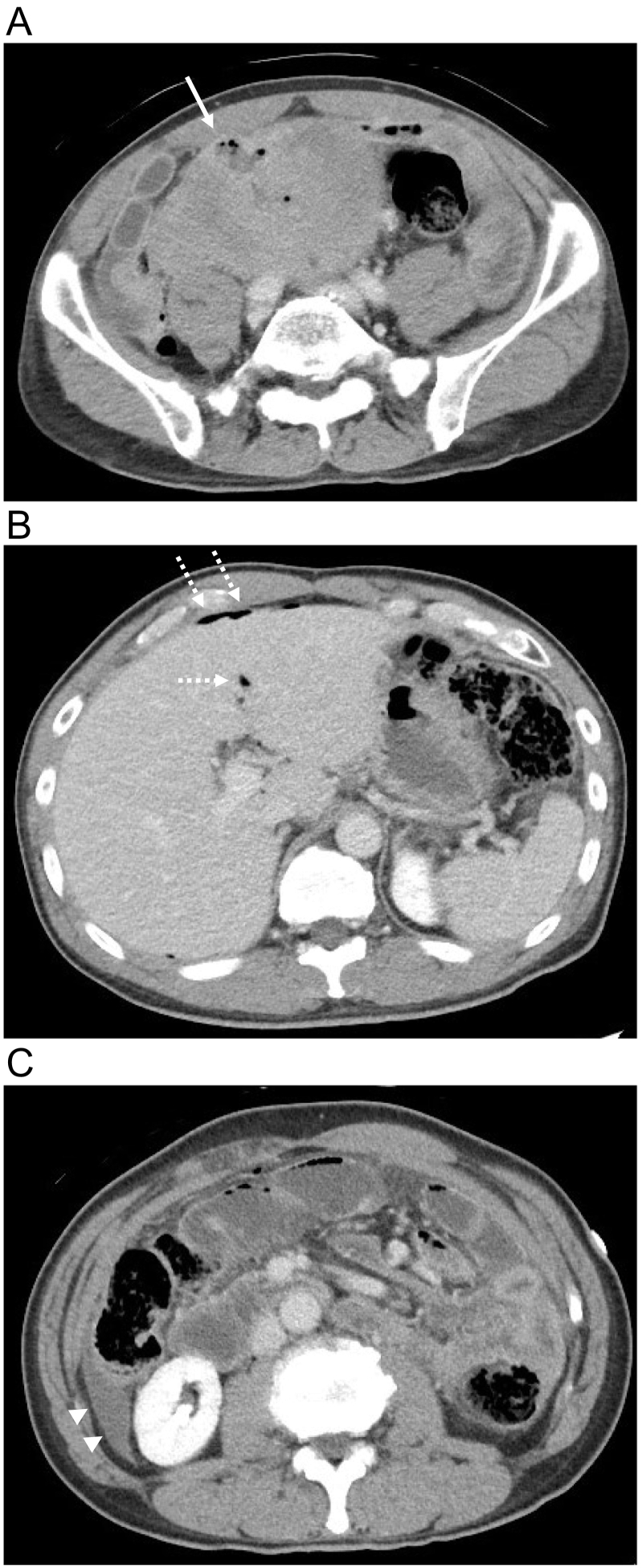
(A) CT revealed a 15 × 15 × 14-cm large internal heterogeneous intra-abdominal tumor with a mosaic-like contrast effect in the pelvis and the lower right abdomen, and abscess formation due to tumor necrosis was present on the ventral surface layer (arrows). Free air (B: dotted arrows) and ascites (C: arrowheads) were found in the abdominal cavity. Although the small intestine appeared enlarged as a whole (C).

The patient was a 57-year-old man. He was emergently admitted to our hospital for lower abdominal pain and distension. There was no notable medical history, such as abdominal surgery, or family history. On admission, the entire abdomen was rigid, and muscle guarding was present, with the most significant tenderness in the lower right abdomen. Blood test findings revealed severe inflammation with a white blood cell count of 1000/μL, C-reactive protein level of 7.25 mg/dL and procalcitonin level of 8.6 ng/mL. Computed tomography (CT) revealed a 15 × 15 × 14-cm large internal heterogeneous intra-abdominal tumor with a mosaic-like contrast effect in the pelvis and the lower right abdomen, and abscess formation due to tumor necrosis was present on the ventral surface layer (Fig. 1A). In addition, free air and ascites were found in the abdominal cavity (Fig. 1B-C), suggesting that this abscess may have ruptured and penetrated the abdominal cavity. Although the small intestine appeared enlarged as a whole (Fig. 1C), there was no definite invasion of the tumor into the intestine.

**Fig. 2:**
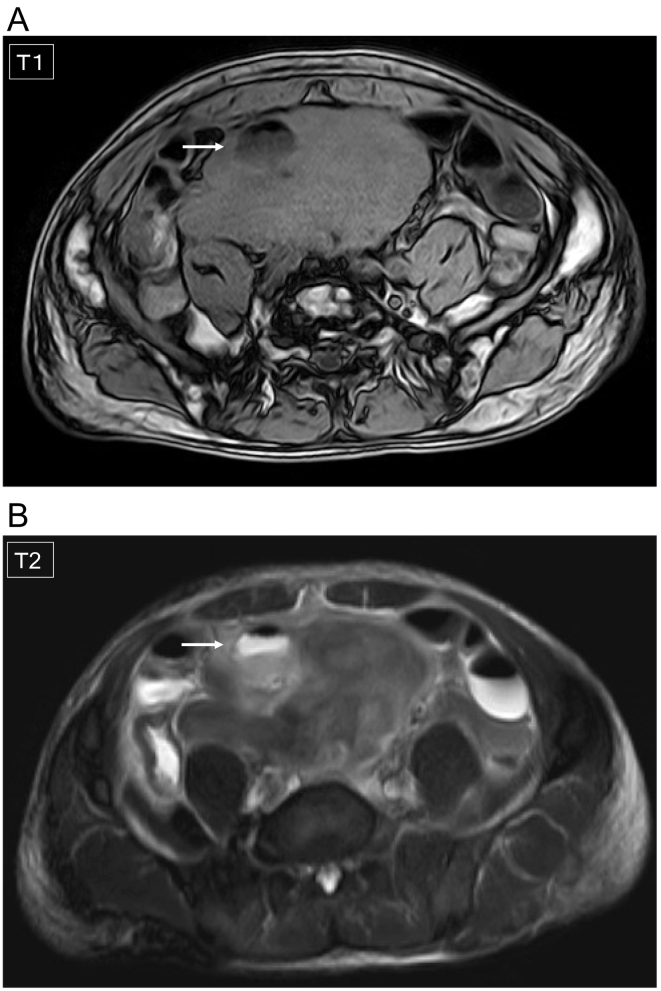
MRI examination revealed that the tumor had an internally non-uniform low signal on both T1- (A) and T2- (B) enhanced images, and fluid component retention was observed in the ruptured abscess (arrows).

Therefore, we diagnosed acute peritonitis due to rupture following intra-abdominal tumor necrosis and intra-abdominal perforation. Since the patient's systematic symptoms were relatively stable, gastrointestinal perforation was ruled out, the differential diagnosis of the tumor itself was difficult at this point, and it was unclear whether or not the tumor could be resected even if emergency surgery was performed, we decided to start with conservative treatment such as fasting and antibacterial administration, and then proceed to differential diagnosis of the tumor, followed by treatment including surgery. When Meropenem 3 g/day was administered after hospital admission, the symptoms of peritonitis were improved the next day. Magnetic resonance imaging (MRI) examination revealed that the tumor had an internally non-uniform low signal on both T1- and T2-enhanced images, and fluid component retention was observed in the ruptured abscess (Fig. 2A-B). The boundary between the tumor and surrounding tissue was relatively clear; we diagnosed that there was no peri-tumor invasion, and that the tumor was therefore resectable. Based on the above results, the preoperative diagnosis was ileocecal gastrointestinal stromal tumor (GIST), or ileocecal malignant lymphoma since the IL-2 receptor level was as high as 1010 U/mL.

Subsequently, there was no intestinal obstruction due to water intake; however, CT re-examination revealed a still dilated small intestine, and there was a concern regarding resuming meals; therefore, laparotomy was performed on the 8th day after hospital admission.

**Fig. 3:**
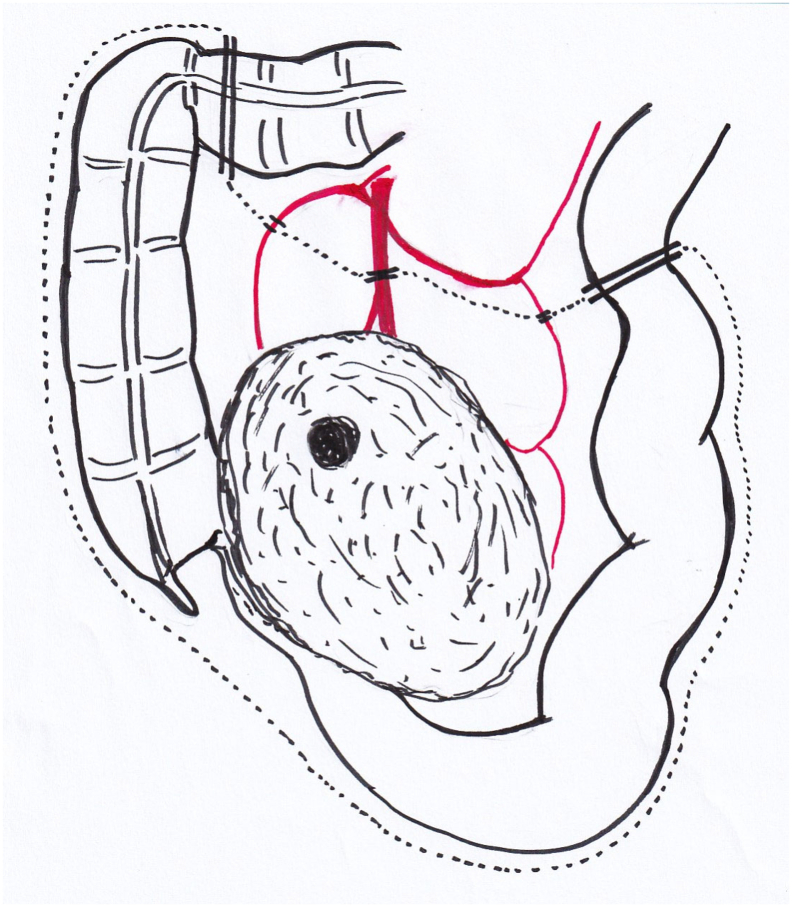
Schema of the tumor localization and resection site of the specimen: The tumor was located in the center of the ileocecal mesentery, and that the terminal ileum was involved in the tumor.

Intraoperative findings revealed that the tumor was located in the center of the ileocecal mesentery, and that the terminal ileum was involved in the tumor (Fig. 3). However, the tumor was relatively easy to remove from the retroperitoneum, and ileocolectomy was performed.

**Fig. 4:**
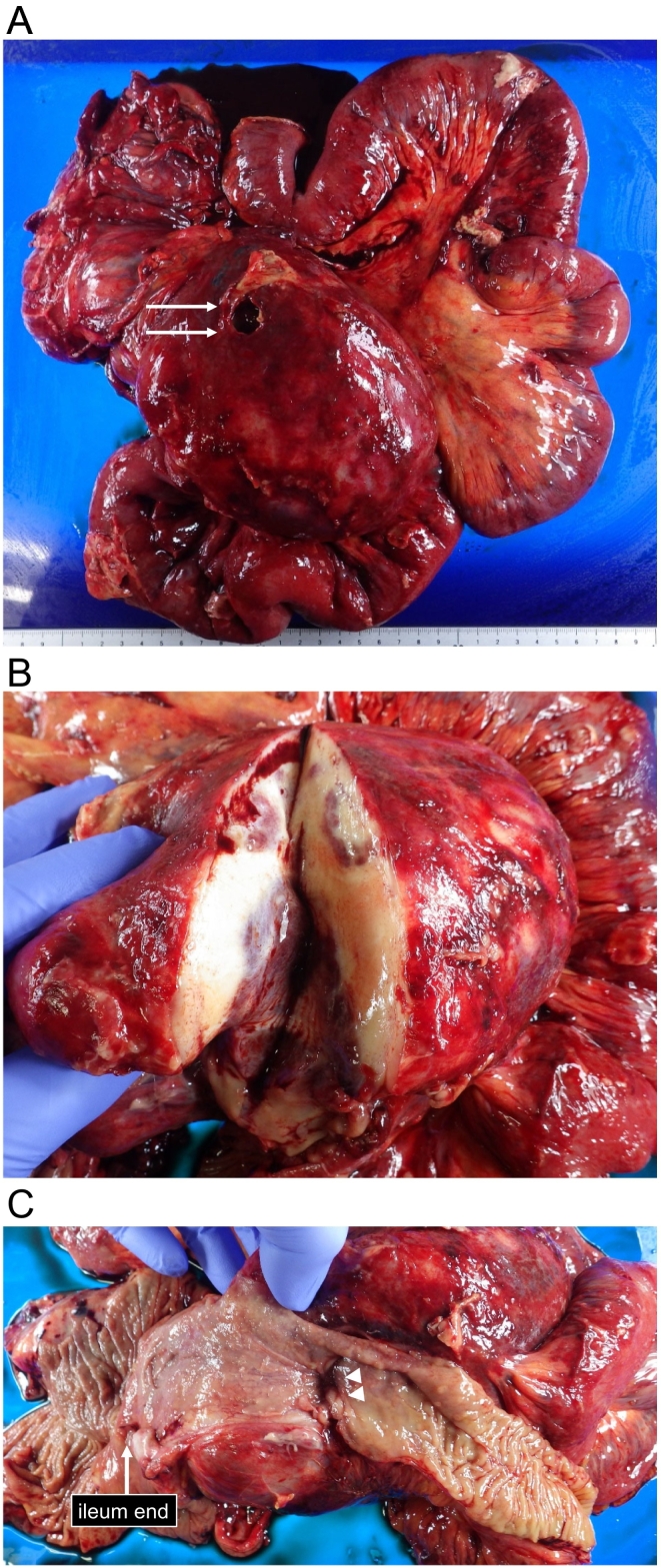
(A) The specimen was a 165 × 125-mm large soft elastic tumor with a necrotic rupture site on the ventral side (arrows). (B) The surface of the tumor section was off-white, and dark red necrotic areas were scattered throughout. (C) The tumor partially involved the wall of terminal ileum and caused luminal stenosis (arrowheads). (For interpretation of the references to colour in this figure legend, the reader is referred to the web version of this article.)

**Fig. 5:**
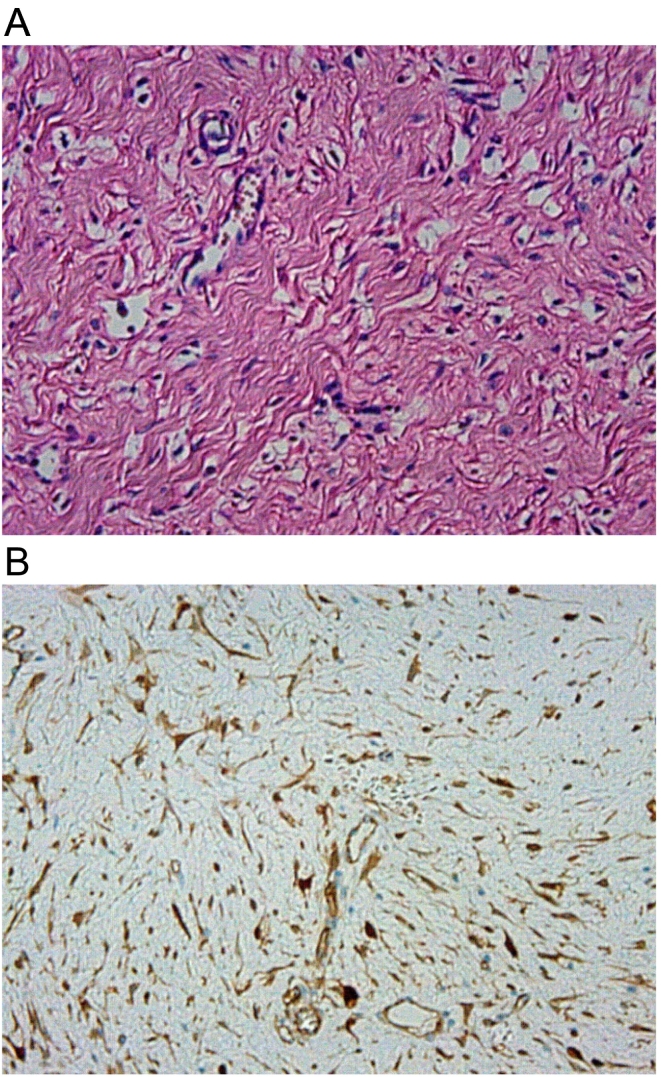
Histopathological examination revealed spindle cells with collagenous fiber hyperplasia (A), and immunohistochemical staining for β-catenin was positive (B).

The specimen was a 165 × 125-mm large soft elastic tumor with a necrotic rupture site on the ventral side (Fig. 4A). The surface of the tumor section was off-white, and dark red necrotic areas were scattered throughout (Fig. 4B). The tumor partially involved the wall of terminal ileum and caused luminal stenosis (Fig. 4C); however, there was no connection between the entrapped intestine and the tumor necrotic rupture site. Histopathological examination revealed spindle cells with collagenous fiber hyperplasia (Fig. 5A), and immunohistochemical staining for β-catenin was positive (Fig. 5B); however, c-kit, CD34, SMA, S100, and desmin were all negative, so we made a diagnosis of MDF.

The postoperative course was uneventful, and he was discharged on the 8th day after surgery (on the 16th day after admission). Since postoperative total colonoscopy found no polyposis, he was denied the diagnosis of familial adenomatous polyposis (FAP). Since complete resection was performed, postoperative adjuvant chemotherapy was not administered. There was no obvious recurrence at 1 year postoperatively.

## 3 Discussion

DF was first reported by MacFarlane in 1833 [5]. DF has several clinicopathological characteristics, including the following: (1) proliferation of highly differentiated fibroblasts; (2) no highly atypical cells or atypical mitotic figures are seen; (3) presence of a large amount of collagen fibers between proliferating cells; (4) infiltrative developmental forms; (5) no distant metastasis, but local recurrence is observed [6]. Generally, it is a type of benign fibroma that does not cause distant metastasis, and is considered to be an intractable disease that invades the surrounding area and repeats local recurrence even if radical surgery is performed.

The mechanism of tumor development is suggested to be associated with 10 to 20% of cases of hereditary diseases, such as FAP and Gardner's syndrome; mechanical stimuli, such as surgery and abdominal trauma; and a history of exposure to radiation appear to be involved as pathogenetic factors in sporadic development [1]. Genetic abnormalities related to tissue repair and dysregulation of connective tissue formation have been considered to be involved [1]. In recent years, it has been pointed out that CTNNB1 gene abnormalities are associated with DF cases, and the nuclear expression of β-catenin can be confirmed by immunostaining [7]. It has been further reported that estrogen receptors are found in 33% of patients with DF, and the involvement of female hormones is also speculated [1]. In our case, there was no history of surgery or trauma, polyposis was not observed on total colonoscopy, so we diagnosed that he was not FAP and Gardner's syndrome.

MDF exhibits few symptoms and is often found after becoming aware of an abdominal mass following tumor growth. However, cases of intestinal obstruction due to tumor invasion, and as in our case, peritonitis after tumor rupture have also been reported [8], [9], [10], [11], [12].

CT and MRI are frequently performed as diagnostic imaging. On MRI, DF appears as a wide low-intensity region on T1- and T2-weighted images and is often seen to have a non-uniform inside; however, sometimes it can present as a high-intensity region on T2-weighted imaging [13]. Meanwhile, MDF exhibits various characteristics depending on the proportion of spindle cells, collagen fibers, and mucous matrix in the tissue; therefore, it is difficult to provide a definitive diagnosis for MDF based on imaging findings when other intra-abdominal tumors, such as GIST, leiomyoma (leiomyoma), malignant lymphoma, and etc. In our case, ileocecal GIST or ileocecal malignant lymphoma was suspected as preoperative diagnosis, but a definitive diagnosis could not be made.

Surgical resection is the first-line treatment for MDF, but it has been reported that MDF recurred in 90% of patients with FAP and 11% of patients without FAP even after complete resection [14], and the postoperative high local recurrence rate is problematic. Consequently, drug therapy and radiation therapy are selected for cases in which radical resection is not possible or for recurrent cases [15]. In drug therapy, non-steroidal anti-inflammatory drugs (meloxicam, indomethacin, sulindac, and celecoxib), anti-estrogenic drugs (tamoxifen and toremifene), cytotoxic chemotherapy (doxorubicin, methotrexate, and vinblastine), and tyrosine kinase inhibitors (imatinib, sunitinib, pazopanib, sorafenib, and sirolimus) have been reported to be effective; however, the number of examined cases is small and sufficient evidence has not been accumulated for most treatment strategies [16], [17], [18], [19], [20].

Although there were two cases in which the MDF itself ruptured to cause peritonitis, emergency surgery was performed in both cases [11], [12]. However, There were no reported cases in which conservative treatment was followed by elective surgery as in our case. The reasons for avoiding emergency surgery on CT were that the size of the ruptured abscess cavity on the tumor surface was limited to some extent, and that possible gastrointestinal perforation and penetration between the ruptured abscess cavity and the gastrointestinal tract was ruled out. All 3 cases underwent complete resection and did not receive postoperative adjuvant therapy, and no symptoms recurred during postoperative follow-up. But, as mentioned above, the local recurrence rate after resection is not low, so it is expected that the optimal treatment at the time of recurrence will be further verified by the accumulation of MDF cases.

## 4 Conclusion

MDF is often treated with surgery after thorough examinations. There are few reports of peritonitis caused by MDF rupture; emergency surgery can be avoided. Although complete resection of the tumor was possible in our case, local recurrence may occur in the future due to tumor rupture of the abdominal cavity, so careful follow-up is essential.

## Funding

This report has not received any funding.

## Ethical approval

This is a case report and it did not require ethical approval from ethics committee according to our institution.

## Consent

Written informed consent was obtained from the patient for publication of this case report and accompanying images. A copy of the written consent is available for review by the Editor-in-Chief of this journal on request.

## Author contribution

Conception and design of the study: MT.

Performed the surgery and perioperative management on the patient: MT, YM, HT.

Data acquisition: MT.

Drafting and revising of the article: MT, YM, TS, HT.

Final approval of the version to be submitted: MT, YM, TS, HT.

## Registration of research studies

NA.

## Guarantor

Masahiro Tawada, MD.

## Provenance and peer review

Not commissioned, externally peer-reviewed.

## Declaration of competing interest

The authors report no conflicts of interest.
